# Supplementary material for: Twenty-Four-Year Trends in Family and Regional Disparities in Fruit, Vegetable and Sugar-Sweetened Beverage Consumption among Adolescents in Belgium
Source: Int J Environ Res Public Health. 2021 Apr 21;18(9):4408. doi: 10.3390/ijerph18094408 (PMC8122606; doi:10.3390/ijerph18094408)
Supplement: Supplementary file 1 [file ijerph-18-04408-s001.zip › SupplementaryMaterials/FigureS2.docx]

**Figure S2.** Prevalence of food consumption by year of survey (HBSC, Belgium, 1990-2002-2014)
