# Supplementary material for: Twenty-Four-Year Trends in Family and Regional Disparities in Fruit, Vegetable and Sugar-Sweetened Beverage Consumption among Adolescents in Belgium
Source: Int J Environ Res Public Health. 2021 Apr 21;18(9):4408. doi: 10.3390/ijerph18094408 (PMC8122606; doi:10.3390/ijerph18094408)
Supplement: Supplementary file 1 [file ijerph-18-04408-s001.zip › SupplementaryMaterials/TableS1.docx]

**Supplementary table 1.** Food consumption and characteristics of participants in percentages by year of survey (HBSC, Belgium, 1990-2002-2014).

|  | **1990**  **n = 8,001** | **2002**  **n = 29,825** | **2014**  **n = 21,939** | ***P*-value** |
| --- | --- | --- | --- | --- |
| **Non-daily fruit** | 27.7 | 69.2 | 60.6 | <0.001 |
| **Non-daily vegetables** | 23.1 | 49.5 | 44.6 | <0.001 |
| **Daily Sugar-sweetened beverages** | 58.9 | 40.7 | 34.8 | <0.001 |
| **Sex** |  |  |  | <0.001 |
| Boys | 47.3 | 48.6 | 51.7 |  |
| Girls | 52.7 | 51.4 | 48.3 |  |
| **Age Group** |  |  |  | <0.001 |
| 10-12 y | 33.6 | 33.5 | 29.6 |  |
| 13-16 y | 45.5 | 47.4 | 49.8 |  |
| 17-19 y | 20.9 | 19.1 | 20.6 |  |
| **Family Structure** |  |  |  | <0.001 |
| Two parents | 83.9 | 77.4 | 65.4 |  |
| Blended family | 4.4 | 9.4 | 14.4 |  |
| Single-parent family | 11.7 | 13.2 | 20.2 |  |
| **School Region** |  |  |  | <0.001 |
| Brussels-Capital | 13.3 | 10.6 | 12.1 |  |
| Wallonia | 36.6 | 37.3 | 47.3 |  |
| Flanders | 50.1 | 52.1 | 40.6 |  |
